# Psychophysiological responses to psychological stress exposure and neural correlates in adults with mental disorders: a scoping review

**DOI:** 10.3389/fpsyt.2023.1191007

**Published:** 2023-07-26

**Authors:** Julija Gecaite-Stonciene, Maria G. Rossetti, Paolo Brambilla, Brian M. Hughes, Narseta Mickuviene, Marcella Bellani

**Affiliations:** ^1^Laboratory of Behavioral Medicine, Neuroscience Institute, Lithuanian University of Health Sciences, Palanga, Lithuania; ^2^Department of Neurosciences, Biomedicine and Movement Sciences, Section of Psychiatry, University of Verona, Verona, Italy; ^3^Department of Neurosciences and Mental Health, Fondazione IRCCS Ca' Granda Ospedale Maggiore Policlinico, Milan, Italy; ^4^Department of Pathophysiology and Transplantation, University of Milan, Milan, Italy; ^5^School of Psychology, University of Galway, Galway, Ireland

**Keywords:** stress, psychophysiology, brain imaging, MRI, psychiatry, review, scoping review, mental disorders

## Abstract

**Introduction:**

The dysregulation of psychophysiological responses to mental stressors is a common issue addressed in individuals with psychiatric conditions, while brain circuit abnormalities are often associated with psychiatric conditions and their manifestations. However, to our knowledge, there is no systematic overview that would comprehensively synthesize the literature on psychophysiological responses during laboratory-induced psychosocial stressor and neural correlates in people with mental disorders. Thus, we aimed to systematically review the existing research on psychophysiological response during laboratory-induced stress and its relationship with neural correlates as measured by magnetic resonance imaging techniques in mental disorders.

**Methods:**

The systematic search was performed on PubMed/Medline, EBSCOhost/PsycArticles, Web of Science, and The Cochrane Library databases during November 2021 following the PRISMA guidelines. Risk of bias was evaluated by employing the checklists for cross-sectional and case-control studies from Joanna Briggs Institute (JBI) Reviewers Manual.

**Results:**

Out of 353 de-duplicated publications identified, six studies were included in this review. These studies were identified as representing two research themes: (1) brain anatomy and psychophysiological response to mental stress in individuals with mental disorders, and (2) brain activity and psychophysiological response to mental stress in individuals with mental disorders.

**Conclusions:**

Overall, the evidence from studies exploring the interplay between stress psychophysiology and neural correlates in mental disorders is limited and heterogeneous. Further studies are warranted to better understand the mechanisms of how psychophysiological stress markers interplay with neural correlates in manifestation and progression of psychiatric illnesses.

## 1. Introduction

Dysregulated psychophysiological responses to psychological stressors is a common feature of people with psychiatric conditions (1–3), signaling the maladaptive coping with daily stressors, which may play a significant role in the etiology of mental disorders (4, 5). The dysregulation of cardiovascular and cortisol stress responses, including hypo- and hyper-reactivity (6) has been extensively studied in laboratory settings in individuals with and without psychiatric conditions, including persons with mood and anxiety disorders (4, 7, 8), trauma- and stressor-related disorders (9), eating disorders (10), personality disorders (11), obsessive-compulsive, and related disorders (12).

Considering the biological mechanisms involved in the development and progression of mental disorders, morpho-functional changes of brain circuits are often linked with psychiatric conditions and their manifestations (13–19), complementing the importance of neuroimaging studies in psychiatry research and clinical application (20). The application of neuroimaging techniques, such as magnetic resonance imaging (MRI) are often used to determine the location and amplitude of morpho-functional changes in the brain linked with mental disorders, ultimately facilitating the identification of potential biomarkers of pathology, to be used to test new therapeutic targets (e.g., neurostimulation techniques, transcranial direct current stimulation, and neurofeedback) (21). Furthermore, mental disorders are also known for functional disruption in neural circuitry underlying emotional processing. For example, both increased and decreased activation of dorsomedial prefrontal cortex during emotional processing of mental image was found in individuals with depression (22) and post-traumatic stress disorder (23). Taking the broader perspective, a recent meta-analysis (24) suggested the dysregulation in the so-called salience network, the ventral striatal and ventromedial prefrontal network as well as lateral orbitofrontal network in those with major mental disorders during emotional processing.

Despite the evident relevance of psychophysiological stress biomarkers and neural correlates in psychiatric conditions, studies examining the interplay between these two groups of biomarkers in clinical populations have been relatively scarce. There have been several narrative reviews on laboratory-induced stress responses and neural correlates in general (25, 26). However, to date, we are not aware of any systematic overview that has mapped or synthesized the body of literature on psychophysiological responses during laboratory-induced mental stressor with that on neural correlates in persons with psychiatric disorders. Examination of such biological variabilities may help bolster our understanding of mechanisms that contribute to the etiology, progression, and manifestation of psychiatric conditions. Thus, the current scoping review aimed to systematically review the existing research on psychophysiological reactions during laboratory-induced stress and their relationship with neural correlates measured by MRI techniques in mental disorders.

## 2. Methods

### 2.1. Search strategy

The strategy used in this review was in line with Preferred Reporting Items for Systematic Reviews and Meta-Analyses (PRISMA) guidelines (27). PubMed/Medline, EBSCOhost/PsycArticles, Web of Science, and The Cochrane Library were chosen as databases to find publications on the selected topic. We also reviewed additional sources, such as clinicaltrials.gov and the GSK Clinical Study Register. The following search query was employed: (“psychophysiolog^*^” OR “cardiovascular” OR “cortisol”) AND “stress” AND (“psychological” OR “acute” OR “mental” OR “social” OR “psychosocial”) AND (“response^*^” OR “reaction^*^” OR “reactivity”) AND (“magnetic resonance imaging” OR “brain imaging” OR “fmri” OR “DTI”) AND (“psychiatr^*^” OR “clinical” OR “patient^*^”).

### 2.2. Selection criteria

The selection criteria were based on PICOS (i.e., population, intervention, comparator, outcomes, study design) approach (28). Publications were considered for inclusion if they (a) were performed in adult in-patient or out-patient psychiatric patients, as defined by any operational defined criteria (e.g., DSM-5, ICD-10) (P); (b) employed standard behavioral method for laboratory-induced mental stress test and structural or functional Magnetic Resonance Imaging (sMRI and fMRI, respectively) for the assessment of morpho-funtional properties of the brain (I); (c) included a healthy control group (C); (d) provided findings on psychophysiological parameters during laboratory-induced stress, such as cortisol response and/or cardiovascular response (e.g., blood pressure, heart rate, heart rate variability, skin conductance etc.) to stress and its association with structural and functional brain indices; (e) were peer-reviewed studies drawing on empirical data (e.g., randomized controlled trials, cross-sectional studies, case-control studies) (S). There were no restrictions to inclusion regarding country of origin, provided that the study was reported in English language.

### 2.3. Review procedure

Relevant studies were retrieved on November 2021 employing the platform of Rayyan Systems Inc. Selected studies were independently reviewed by two assessors (JGS and MGR). Inclusion and exclusion criteria were determined beforehand. After initial screening of title and abstracts, the assessors reviewed full-text publications, which were retrieved and independently verified for eligibility. Disagreements were acknowledged and resolved through discussions and unified consensus, or with revision of a third senior assessor (MB). Data that was retrieved from publications were analyzed taking a narrative synthesis approach.

### 2.4. Risk of bias (quality) assessment

Two independent reviewers (JGS and MGR) performed critical appraisal by using Joanna Briggs Institute (JBI) Reviewers Manual (29, 30), a standardized quality assessment tool for clinical (cross-sectional/case-control) studies (31).

## 3. Results

### 3.1. Included studies

**Figure 1:**
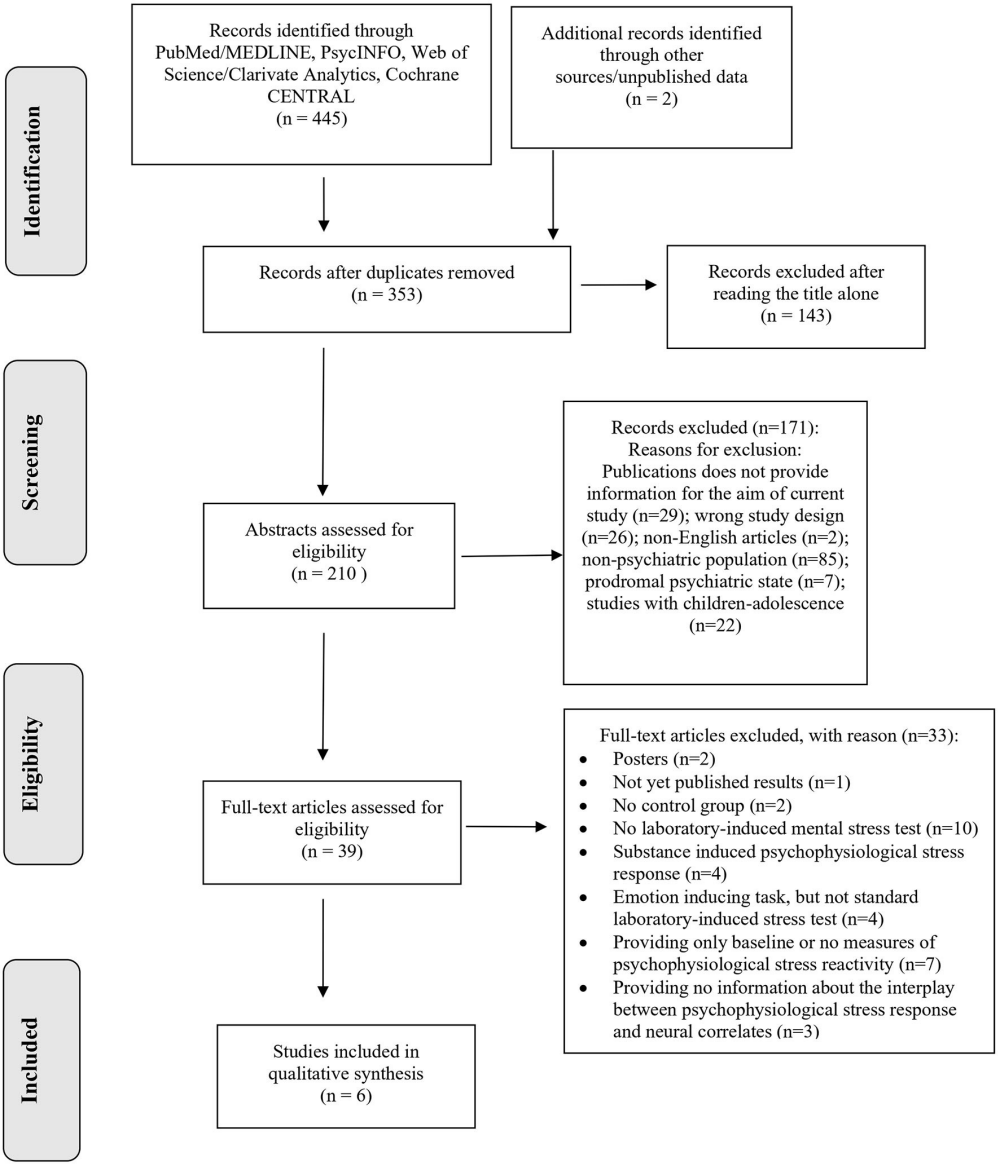
PRISMA 2009 flow chart of study selection.

A flow diagram illustrating the studies selection process is presented in Figure 1. The systematic search retrieved 353 de-duplicated records. After title and abstract screening, 314 records were excluded because they clearly did not meet the inclusion criteria. A total of 39 studies underwent for full-text review, after which six studies (32–37), were identified for inclusion in this scoping review and critically appraised in a descriptive manner. Two themes were identified, namely (a) brain anatomy and psychophysiological response to laboratory-induced mental stress in individuals with psychiatric disorders, and (b) brain activity and psychophysiological stress response in individuals with psychiatric disorders.

### 3.2. Risk of bias within studies

Overall, bias in study methodology was low. However, most of the studies lacked information on confounding variables and/or statistical analysis, while controlling for possible covariates (e.g., sociodemographic, clinical data and/or disorder specific characteristics). Whilst two studies addressed the possible confounding factors (34, 35), the other four studies did not provide the information and statistical results on confounding (32, 33, 36, 37).

### 3.3. Study synthesis

The studies were published between 2003 and 2020, of which three (32–34) were conducted in North America, two in Europe (36, 37), and one in Asia (35). Several mental health conditions were investigated, including antisocial personality disorder (APD) (32), posttraumatic stress disorder (PTSD) (34), major depressive episode (MDE) (35), bipolar disorder (BD) (36), obsessive-compulsive disorder (OCD) (33), and Cannabis Use Disorder (CUD) (37). All samples were single-sex, with the sample sizes ranging from 39 (33) to 105 (35). To measure neural correlates, the majority of the studies (*n* = 4) used fMRI (33, 35–37), out of which three studies included fMRI scan during the resting period (33, 35, 37). The remaining two out of six studies employed sMRI (32, 34). As a laboratory induced mental stress test, two studies employed Trier Social Stress Test (TSST) (34, 36), which implements public speech and arithmetic tasks as psychological stressors (38). Three studies employed Montreal Imaging Stress Task (MIST) (33, 35, 37), which is derived from TSST, yet contains only computerized mental arithmetic task (39). Lastly, a single study used solely regular public speaking task (32). The detailed characteristics of each study are outlined in Table 1.

**Table 1:** Descriptive information of selected studies.

**References**	**Country**	**Type of study**	**Study population (sample size, *n*)**	**Sex [*n* (%) women]**	**Mean age (SD)**	**Imaging technique**	**Imaging outcome measures**	**Stress test**	**Biomarker for stress psychophysiology**	**Psycho-DIAGNOSTICAssessment**	**Other variablesmeasured**	**Medication**	**Main results**
Raine et al. (32)	USA	Case-control study	Community sample (*n* = 83) out of which: APD group (*n* = 15); matched HC (*n* = 25)	83 (0 %)	APD group: 31.6 (6.6) HC:28.8 (6.5)	sMRI	Functional callosal measures; structural callosal properties (thickness; volume, area)	Public speech	Autonomic activity (i.e., skin conductance and heart rate)	SCID-I SCID-II	Psychopathy traits; IQ; history of head injury; social status; self-reported crime and criminal history; substance dependence; social functioning	N/A	↑ Corpus callosum volumes in APD group vs. HC; Associations between ↑callosal volumes and ↓ autonomic stress reactivity (i.e., reduced skin conductance and lower heart reactivity during the stressful task) in community sample.
Lord et al. (33)	Canada	Cross-sectional study	Women with postpartum OCD (*n* = 12); postpartum HC (*n* = 16), healthy mothers (*n* = 11)	39 (100 %)	Women with postpartum OCD: 33.0 (5.1); postpartum HC: 31.9 (4.6); healthy mothers: 34.8 (6.0)	fMRI	Whole-brain analysis	MIST	Salivary cortisol	OCD diagnosis determined by psychiatrist; CIDI-VENUS; Y–BOCS; POCS	Depressive and anxiety symptoms; experience of childhood trauma; subjective sleep quality	Medication use is present, not specified	Postpartum OCD vs. postpartum HC and healthy mothers: ↑cortisol response to MIST; positive correlation between MIST-related cortisol level and OCD/temporal cortices.
Babson et al. (34)	USA	Cross-sectional study	Military veterans: with PTSD (*n* = 45), without PTSD (*n* = 45)	90 (0%)	With PTSD: 49.11 (87.96); Without PTSD 47.11 (10.56)	sMRI	Volumes of rostral and caudal anterior cingulate cortex, parcels, hippocampus, and amygdala; supratentorial, ACC, hippocampal, and amygdala volumes; the sum of gray and white matter within delineated volumes	TSST	Salivary cortisol	SCID-I, clinician-administered PTSD scale	Comorbid psychiatric psychopathologies	Use of psychotropic medications, not specified (controlled effects)	In the whole group (*n* = 90): the association between ↑global cortical volume and ↑cortisol response during TSST; no comparisons between PTSD group vs. HC.
Ming et al. (35)	China	Cross-sectional study	First MDE patients (*n* = 36); rMDE patients (*n* = 33); HC (*n* = 36)	105 (100%)	First MDE patients: 22.81 (4.25) rMDE patients: 21.67 (3.14); HC: 22.19 (1.60)	fMRI	Whole-brain analysis of Blood oxygen-level-dependent response	MIST	Salivary cortisol	SCID-I	History of childhood trauma, depressive symptoms, subjective stress levels.	No medications	Stress-induced vmPFC activation changes linked with ↑ cortisol response during mental stress challenge in all the three groups. rMDE/MDE vs. HC: ↑cortisol response and ↓vmPFC and ↑precuneus, cingulate and paracentral lobule.
van Leeuwen et al. (36)	The Netherlands	Cross-sectional study	HC group-no TSST (*n* = 19) HC group with TSST (*n* = 20) BD group-no TSST (*n* = 19) BD group with TSST (*n* = 19)	80 (0 %)	HC group-no TSST: 41.5 (1.7) HC group with TSST: 39.0 (1.8) BD group-no TSST: 42.9 (1.8) BD group with TSST: 39.8 (2.0)	fMRI	Ventral striatum and OFC blood oxygen level–dependent BOLD response	TSST	Salivary cortisol	IDS-C; YMRS; MINI	Life stressors	Absence of corticosteroids and antipsychotic drugs, other drugs not specified	In HC only: ↑ventral striatal responses after TSST; no significant associations between striatal or OFC responses and salivary cortisol response (comparisons HC vs. BD groups not reported)
Zhao et al. (37)	Germany	Cross-sectional study	CUD (*n* = 28) HC (*n* = 23)	51 (0 %)	CUD: 25.54 (5.11) HC: 24.57 (3.55)	fMRI	Precuneus/middle occipital gyrus; insula/middle frontal gyrus; middle frontal gyrus/medial frontal gyrus/cingulate gyrus; middle frontal gyrus.	MIST	Cardiovascular stress response (i.e., blood pressure and heart rate)	MINI	Depressive symptoms, Anxiety symptoms, mood changes, attention, Nicotine use, Subjective stress levels	No use of medications	CUD vs. HC: ↓cardiovascular stress reactivity in the cluster in the right precuneus. ↓cardiovascular stress reactivity in those with CUD linked with ↑connectivity of this region with the superior frontal gyrus

APD, antisocial personality disorder; BD, bipolar disorder; CAPS, clinician-administered PTSD scale; CIDI-VENUS, composite international diagnostic interview for women; CUD, cannabis use disorder; DSM-IV, diagnostic and statistical manual of mental disorders, 4th edition; HC, healthy controls; IDS-C, inventory of depressive symptomatology, clinician rating; MDD, major depressive disorder; MDE, major depressive episode; MINI, Mini-International neuropsychiatric inter-view; MIST, Montreal Imaging Stress Task; OCD, obsessive compulsive disorder; OFC, orbitofrontal cortex; POCS, perinatal obsessive-compulsive scale; PTSD, post-traumatic stress disorder; rMDD, recurrent major depressive disorder; rMDE, remitted major depressive episode; SCID-I, structured clinical interview for axis I DSM-IV disorders; SCID-II, structured clinical interview for axis II personality disorders; sMRI/fMRI, structural/functional magnetic resonance imaging; TSST, trier social stress test; vmPFC, ventromedial prefrontal cortex; Y-BOCS, Yale–Brown obsessive compulsive scale; YMRS, Young Mania Rating Scale.

#### 3.3.1. Studies of psychophysiological responses to mental stress in individuals with mental disorders that focused on brain anatomy

Two studies examined the interplay between brain structure and psychophysiological stress response (32, 34). Both studies used sMRI, while the measures of psychophysiological response to stress and the employed protocol to observe it considerably differed. The laboratory-induced stress tasks and sMRI were conducted consecutively.

The case-control study by Raine et al. (32) evaluated 40 men within a community sample of *n* = 83, of which 15 individuals met the criteria for APD as measured with Structured Clinical Interview for Axis II Personality Disorders (SCID-II), and 25 were matched healthy controls (HC). The primary interest was to evaluate the callosal abnormalities and its manifestation in those with APD. Additionally, the authors also measured how callosal abnormalities were associated with emotional deficits, including reduced autonomic stress responses. To measure autonomic reactivity (i.e., skin conductance and heart rate) to stress, a public speaking task was performed, during which the participants had to prepare (2 min.) and talk (2 min.) about their worst faults (40). Correlational analysis suggested the associations between larger callosal volumes and low autonomic stress reactivity (i.e., reduced skin conductance and lower heart reactivity during the stressful task), providing the results on blunted affect and its interconnectedness with brain structure in APD. Of note, this additional correlational analysis was performed in the larger (*n* = 83) community sample, in which APD-related characteristics were assessed via the Psychopathy Checklist (41) but the diagnosis of APD was not investigated, while no comparisons between APD and HC were performed. Nevertheless, the major interpretation of the results was that abnormal interhemispheric connectivity might partly account for autonomic blunting during social stressor in those with APD.

Further, the cross-sectional study (34) evaluated 90 military veterans (all men), including 45 individuals with PTSD as measured with Structured Clinical Interview for Axis I DSM-IV Disorders (SCID) and 45 HC. Most individuals with PTSD had a comorbid diagnosis of Major Depressive Disorders. The Trier Social Stress Test (TSST) was employed to measure cortisol response during mental stress challenge. The primary aim was to evaluate regional brain volumes and salivary cortisol measures (i.e., basal, during psychological stress, and DEX-suppressed). The study found that the estimates of global cortical volume but not hippocampal or amygdala volume were moderately linked with cortisol response during the stressful tasks of TSST, while performing the analysis in the whole group (*n* = 90). The results remained significant even when controlled for PTSD diagnosis, smoking history, alcohol use and study site.

#### 3.3.2. Studies of psychophysiological responses to mental stress in individuals with mental disorders that focused on brain activity

We found four studies (33, 35–37) that evaluated the association between brain activation and psychophysiological stress response (i.e., cortisol and cardiovascular responses) in people with vs. without mental disorders. While one study performed laboratory-induced stressful task followed by fMRI scan (36), others did both tasks simultaneously (33, 35, 37).

Two cross-sectional studies focused on affective disorders, studying individuals with current or remitted MDE (MDE and rMDE, respectively) (35) or bipolar disorder (BD) (36). The first study, conducted in China by Ming et al. (35), investigated women with first MDE (*n* = 36), rMDE (*n* = 33), and HC (*n* = 36) with the primary aim to examine state-independent (trait) and state-dependent neural responses to psychological stress in the study participants. Psychiatric diagnosis was confirmed with administration of SCID-I. The MIST was adapted to elicit psychological stress during fMRI. When compared with HC, both rMDE and MDE groups showed higher stress-related cortisol response and similar brain activations, including lower activation of ventromedial prefrontal cortex and greater activation of precuneus, cingulate, and paracentral lobule. Regarding the interplay between neural correlates and psychophysiological stress responses, it was found that stress-induced changes in ventromedial prefrontal cortex activation was inversely associated with higher cortisol response during mental stress in all three groups. According to the authors, the results highlighted the importance of the medial prefrontal cortex in regulation of hypothalamic-pituitary-adrenal axis stress reactivity. These results may possibly explain that the differences found between HC and MDE/rMDE are more attributable to trait effect of depression rather than state or clinical status of onset/remission of depression.

In the second study, conducted in the Netherlands (36), 80 men were studied, of whom 38 had BD as determined by clinician-rated scales (Table 1). The primary aim was to explore ventral striatal and orbitofrontal cortex (OFC) responses during a reward processing task right after TSST in euthymic BD patients vs. HC. Cortisol responses were evaluated during the TSST, while fMRI was conducted together with the reward task. The findings showed no significant associations between striatal or OFC responses and salivary cortisol response during TSST in any of the groups and higher striatal responses after TSST only in HC sample. Comparisons between HC and BD groups were not reported. Overall, the findings suggested altered recovery from psychological stress in individuals with BD regarding striatal reward processing. According to the authors, their findings of reduction of stress-related dynamics in reward processing could partially explain an amplified sensitivity for repeated mood episodes right after acute distressful experiences.

One cross-sectional study (33), conducted in Canada, compared 12 women with postpartum OCD, as determined by study psychiatrists, 16 healthy mothers within their first 6 months postpartum (henceforth labeled as “postpartum HC”) and 11 HC with more than 1 year postpartum (henceforth labeled as “healthy mothers”). The primary aim was to evaluate the cerebral and endocrine measures of psychological stress reactivity during the postpartum period and in the presence of OCD. They all underwent laboratory-induced MIST, while simultaneously being scanned with fMRI. In comparison to postpartum HC, those with postpartum OCD showed heightened cortisol response to mental stress and this was associated with distinct brain activation pattern during MIST, including greater activation in OFC and temporal cortices. The findings suggest that OCD is linked with increased activity within brain regions that are related to alertness, threat detection and emotional liability. It is also suggested that healthy postpartum mothers might have more adapted emotional response to psychological stress due to no activation in OFC in comparison to postpartum women with OCD that showed increased activation of OFC.

Finally, a cross-sectional study conducted in Germany (37) investigated 51 men, including 28 individuals with CUD, as confirmed with the administration of MINI Neuropsychiatric Interview (42) and 23 HC. The authors aimed to identify the integrity of behavioral and neural stress reactivity in those with CUD. Cardiovascular stress responses (i.e., blood pressure and heart rate) were evaluated during MIST and simultaneous fMRI acquisition. The major results suggested that, compared to HC, participants with CUD exhibited decreased cardiovascular stress reactivity in the cluster in the right precuneus. It also suggested that decreased cardiovascular stress reactivity (i.e., blood pressure and heart rate) in those with CUD correlated with increased connectivity between precuneus and the superior frontal gyrus, suggesting the importance of acute stress-induced cognitive performance deficits in those with CUD.

## 4. Discussion

In this current scoping review, we identified six studies examining psychophysiological response to laboratory-induced stress and neural correlates, measured with MRI. Two themes were identified, including (1) brain anatomy and psychophysiological response to mental stress in individuals with mental disorders, and (2) brain activity and psychophysiological response to mental stress in individuals with mental disorders. With regards to brain anatomy, there is some limited knowledge on callosal abnormalities and autonomic stress reactivity in individuals with APD and regional brain volumes and cortisol stress response in those with PTSD. In terms of brain activity, there is some evidence from observational studies, investigating ventromedial prefrontal cortex activation, ventral striatal responses, and cortisol response in affective disorders as well as cerebral correlates and cortisol stress response in postpartum women with OCD.

### 4.1. Appraisal of the identified themes by the present research

The first two studies (32, 34) investigated brain anatomy and its interplay with either autonomic nervous system activation of hypothalamic pituitary-adrenal (HPA) axis activation during mental stress challenge in either men with APD (32) or PTSD (34). In the first study (32) larger callosal volumes were associated with lower autonomic stress reactivity in those with APD, while in the second study (34) the presence of PTSD did not play a role in the interplay between neural and endocrine correlates during psychological stress. The latter study somewhat contradicted the results found in healthy young men (43) where larger hippocampal volume was significantly linked with higher cortisol response during TSST. However, in the study exploring individuals with PTSD (34) the possible underlying reasons were not discussed, which might be linked with reduced hippocampal volume in individuals with PTSD (44). Unfortunately, even though both studies presented the results on psychophysiology of stress (i.e., skin conductance, heart rate and cortisol responses) and neural correlates (i.e., callosal abnormalities and regional brain volumes) corresponding to the brain anatomy, the methodological differences between the studies limits the extent to which their results can be meaningfully compared. In addition, Raine et al. (32) conducted their correlational analysis not specifically on participants who met criteria for APD (*n* = 15), but on the wider community-based sample from which this target group was drawn (*n* = 83). Thus, attributing those results to clinical APD should be done cautiously. It is also important to note that the sample of both studies included men only.

Additional four studies found associations between brain activity (e.g., ventromedial prefrontal cortex activation, ventral striatal responses or cerebral correlates or precuneus activity) and psychophysiological responses (e.g., cortisol or cardiovascular response) to stress in women with remitted MDE (35), men with BD (36), women with postpartum OCD (33), and men with CUD (37). In most of the studies (three out of four) stress-induction paradigms and fMRI were performed simultaneously (33, 35, 37), while showing the significance of acute stress on different brain activity measures in mental disorders. However, differences between studies in the methodological approach prevent from a direct comparisons making them highly heterogeneous and lacking common biological mechanisms.

Regarding further studies, based on the results of this scoping review, it is evident that there is a need for more interdisciplinary research of stress psychophysiology and brain imaging in people with mental disorders. Most mental disorders are currently still under-represented within the research of neuroimaging and stress psychophysiology, even though brain changes and psychobiology of stress in mental disorders are known to be altered relative to healthy populations (1, 13–16). One of the reasons for the scarcity of studies could be the need for relatively expensive and complex scientific investigations. Secondly, all the studies have employed rather small sample sizes that are homogeneous in terms of gender. Thus, further studies, investigating larger samples and examining sex differences could help to provide a better understanding of any role of gender that might be attributed to the interplay between stress psychophysiology and brain anatomy or activity, given that sex/gender often plays a cardinal role in psychiatric psychopathology (45). Finally, longitudinal and/or experimental studies need to be conducted, as we did not find any studies that could definitively assess the causal relationship between psychophysiological stress responses and neural correlates.

### 4.2. Limitations

The limitations of this scoping review are worth mentioning. We found a small number of studies, precluding us from making systematic comparisons across the research or from providing a broader map on the existing literature within this topic. This overview was also limited to only English-language articles, thus excluding potentially relevant studies. Further, due to high heterogeneity of the studies, the comparison between studies were challenging. Specifically, it was difficult to disentangle what findings were determined by disorder specific reasons and which one depicted the unique and transdiagnostic interplay between neural and cardiovascular/endocrine correlates. In fact, high levels of heterogeneity could be observed not only regarding the mental disorders, but also types of stress test (e.g., MIST, TSST), biomarkers (e.g., cardiovascular measures, salivary cortisol, skin conductance), sMRI brain structures (e.g., white matter, gray matter) or fMRI modalities (resting fMRI, reward task). However, in terms of the strengths, this review used transparent, systematic, and rigorous methods throughout the entire process, employing a broad search of the literature with four different databases, and ensuring a good quality of the selected studies with additional quality assessment.

## 5. Conclusion

Our scoping review provided a critical systematic overview of the existing empirical research on psychophysiology of laboratory induced-stress and neurobiological measures during magnetic resonance imaging in psychiatric in-patient and out-patient populations. The selected studies provided further knowledge of the importance to study neural correlates and its interplay with stress psychophysiology in individuals with mental disorder. However, many limitations exist related to the methodology and heterogeneity of the studies. Further research may concentrate on larger study samples, employing more diversity in terms of mental disorders and sex. Also, longitudinal studies are still warranted, allowing to draw the causal relationship between stress psychophysiology and brain circuit abnormalities.

## Data availability statement

The original contributions presented in the study are included in the article/supplementary material, further inquiries can be directed to the corresponding author.

## Author contributions

JG-S: conceptualization and writing—original draft. MGR: conceptualization and writing—review and editing. PB and MB: conceptualization and supervision. BH and NM: writing—review and editing and supervision. All authors contributed to the article and approved the submitted version.
